# Supplementary material for: Conditional chemoconnectomics (cCCTomics) as a strategy for efficient and conditional targeting of chemical transmission
Source: eLife. 2024 Apr 30;12:RP91927. doi: 10.7554/eLife.91927 (PMC11060718; doi:10.7554/eLife.91927)
Supplement: Figure 3—source data 3. [file elife-91927-fig3-data3.zip › Figure 3 source data3 part 1.zip!/Figure 3-Source data 3-part1/1175-9-CRISPResso_Report_rtnd62ko.zip!/CRISPResso_on_1175_11/9.Alleles_frequency_table_around_sgRNA_gtaggccacgcagtagacca.pdf]

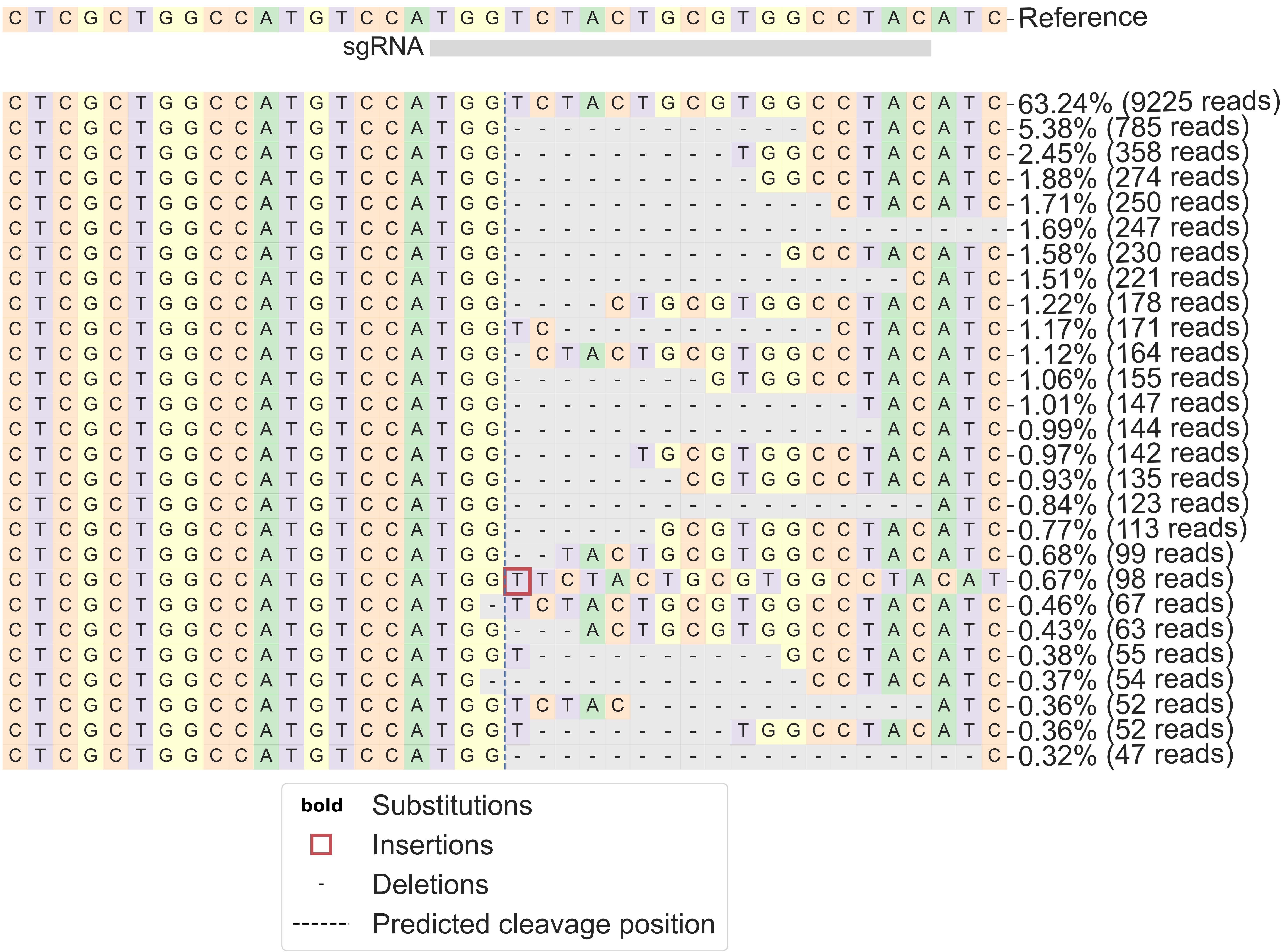

Reference
sgRNA
63.24% (9225 reads)
5.38% (785 reads)
2.45% (358 reads)
1.88% (274 reads)
1.71% (250 reads)
1.69% (247 reads)
1.58% (230 reads)
1.51% (221 reads)
1.22% (178 reads)
1.17% (171 reads)
1.12% (164 reads)
1.06% (155 reads)
1.01% (147 reads)
0.99% (144 reads)
0.97% (142 reads)
0.93% (135 reads)
0.84% (123 reads)
0.77% (113 reads)
0.68% (99 reads)
0.67% (98 reads)
0.46% (67 reads)
0.43% (63 reads)
0.38% (55 reads)
0.37% (54 reads)
0.36% (52 reads)
0.36% (52 reads)
0.32% (47 reads)
bold Substitutions
Insertions
Deletions
Predicted cleavage position
